# 651. Variation in Adult Burn Resuscitation Protocols Across ABA-Verified U.S. Burn Centers: A Cross-Sectional Review

**DOI:** 10.1093/jbcr/irag033.488

**Published:** 2026-04-06

**Authors:** Lud H Eyasu, Megan E Daniels, Kealani Unkel, Jatin Vemuri, Rendell Bernabe, Zachary Brown, Prabhu Senthil-Kumar, Michael J Feldman

**Affiliations:** Evans-Haynes Burn Center at VCU Medical Center/Virginia Commonwealth University, Richmond, VA; Evans-Haynes Burn Center at VCU Medical Center/Virginia Commonwealth University, Richmond, VA; Evans-Haynes Burn Center at VCU Medical Center/Virginia Commonwealth University, Richmond, VA; Prisma Health/University of South Carolina School of Medicine, Richmond, VA; Evans-Haynes Burn Center at VCU Medical Center/Virginia Commonwealth University, Richmond, VA; Evans-Haynes Burn Center at VCU Health, Richmond, VA; Evans-Haynes Burn Center at VCU Medical Center/Virginia Commonwealth University, Richmond, VA; Virginia Commonwealth University, Richmond, VA

## Abstract

**Introduction:**

Adequate early fluid resuscitation remains the cornerstone of survival after major thermal injury, but excessive or insufficient resuscitation contributes to mortality and morbidity, including renal failure, pulmonary complications, and abdominal compartment syndrome. Despite the presence of national guidelines presented by the American Burn Association (ABA), variability still exists in center-level resuscitation practices. This study aimed to analyze the protocol variation among ABA-verified centers to inform standardization efforts.

**Methods:**

We conducted a cross-sectional review of adult ABA-verified U.S. burn centers. Protocols were obtained from public websites and through direct requests to program leadership. For each center, we assessed the protocol for the following variables: initial resuscitation formula, fluid type, timing and use of colloids, titration endpoints (e.g., urine output), and use of decision-support tools.

**Results:**

Protocols were obtained from 66.2% (43 of 65) of ABA-verified adult burn centers. The most common initial resuscitation formula was the Modified Brooke formula (48.8%), followed by 3 mL/kg/hr (14.6%) and the Parkland formula (12.2%), while 24.4% of centers used other strategies. Lactated Ringer’s solution was specified in nearly all protocols (97.6%), with only 2.4% using variable crystalloids based on Total Body Surface Area (TBSA) and provider preference. All centers monitored urine output (UOP) for titration, with targets of 30–50 mL/hr in 53.9% of protocols, approximately 30 mL/hr in 17.9%, and 0.5–1.0 mL/kg/hr in 10.3%. Colloid administration was included in many protocols, with albumin specified in 72.5% and fresh frozen plasma (FFP) in 32.5%, typically after high crystalloid requirements or in cases of electrical injury. Computer-based decision-support tools, such as Burn Navigator, were rarely used, appearing in only 9.5% of protocols; most centers relied on worksheets with calculations performed by nursing or physician staff.

**Conclusions:**

Substantial variability exists among ABA-verified centers in the choice of resuscitation formulas, fluid types, and titration endpoints. Recognizing shared practices and key differences can support the development of consensus guidelines and help reduce inconsistencies in burn resuscitation. Future studies should aim to understand the rationale behind the use of different protocols across centers, with the goal of incorporating these insights into the development of standardized protocols that promote widespread adoption and uniformity.

**Applicability of Research to Practice:**

Mapping current protocols helps clinicians benchmark local practice, highlights opportunities to combat over- or under-resuscitation, and supports the adoption of shared targets and decision-support tools.

**Funding for the study:**

N/A.

